# All-Hazards Vulnerability and Adaptation Assessment of Canadian Kidney Care Systems: Protocol for a Qualitative Study

**DOI:** 10.2196/90059

**Published:** 2026-04-23

**Authors:** Saly El Wazze, Shinjini Mondal, Shaifali Sandal

**Affiliations:** 1 Division of Nephrology, Department of Medicine, McGill University Health Centre, 1001 Boul Decarie D05-7160, Montreal, QC, H4A 3J1, Canada, 1 5149341934

**Keywords:** vulnerability and adaptation assessment, disasters, emergencies, nephrology, dialysis, Canada, patients

## Abstract

**Background:**

Health and health care systems are progressively challenged by more frequent natural and human-caused hazards. A hazard becomes a disaster when vulnerability and exposure interact in complex ways with system capacity to trigger major disruptions in the functioning of a community or society. Patients with kidney diseases, especially those on dialysis, are particularly vulnerable.

**Objective:**

We will conduct a vulnerability and adaptation assessment (VAA) of Canadian provincial health systems and individual kidney care networks (KCNs) with respect to natural and human-caused disasters and emergencies. Our research aims to map existing KCNs across Canada, contextualize the current and future vulnerability of health systems and patients to disasters and emergencies, and identify and prioritize adaptation measures in partnership with key interest holders.

**Methods:**

We are conducting a bottom-up VAA following a qualitative descriptive approach and using the framework previously developed as an operational guide. We started by conducting an environmental scan and document review and identified 75 KCNs across Canada. After collecting directives and policies on disaster management from these KCNs, we will conduct a content analysis guided by our framework in disaster management. We are now conducting semistructured interviews with kidney health care professionals across Canada recruited using purposive and snowballing techniques to better understand their perspectives, expertise, and lived experiences. We will conduct thematic analysis using an inductive-deductive approach guided by the framework. Findings from these multiple data sources will then be triangulated to generate a robust VAA.

**Results:**

We want to identify the existing operational and human vulnerabilities and the risks associated, as well as explore lessons learned, to develop needed adaptations. Emerging data reflect a variety of experiences across programs and provinces. The findings will explore analysis at the program, provincial, and health system levels. The environmental scan was initiated in November 2024, and the semistructured interviews started in February 2025. We have recruited 71 participants from 55 KCNs. Interviews are ongoing, and coding has been initiated simultaneously. The first VAA focusing on in-center hemodialysis services is expected to conclude in April 2026. These findings are projected to be written up by May 2026, and manuscripts are expected to be submitted for publication to peer-reviewed journals. Thereafter, similar analyses will be conducted focusing on other kidney replacement therapies and pediatric programs.

**Conclusions:**

This is the first VAA of KCNs across Canada. Findings will provide a critical foundation for understanding vulnerability, supporting strategic planning, and guiding adaptation measures that can strengthen health system resilience and mitigate associated risks of disasters and emergencies to patients with kidney diseases.

## Introduction

### Background

More frequent hazards are progressively challenging health and health care systems [1,2]. Hazards can be natural (earthquakes, tsunamis, cyclones, extreme temperatures, and floods), biological (disease outbreaks), technological (explosions and infrastructure failures), or societal (conflict, wars, and humanitarian emergencies) [3]. Their impact reflects the interplay between the exposure and the vulnerability of the affected population [4-7]. A hazard becomes a disaster when these 2 factors interact in complex ways with system capacity to trigger a major disruption in the functioning of a community or society [8]. In contrast, emergencies involve hazardous events that do not precipitate large-scale societal disruption [8]. Several populations are more vulnerable to the impacts of these disasters and emergencies, and patients with kidney diseases in particular experience a disproportionately higher risk with hazard exposure [9].

The survival of those with end-stage kidney disease who receive dialysis depends on the safe provision of this therapy multiple times a week [10-12]. Receiving dialysis is dependent on intact and functioning infrastructure, the availability of medical personnel, and the supply of medical equipment and medications, all of which can be disrupted during a disaster or emergency setting [11,13-25]. Surveys of health care providers such as physicians, nurses, and technicians have reported challenges they experienced in getting to a dialysis center and providing services during or following disasters [20,22-24]. Several studies have reported disruptions in dialysis care delivered to patients in such settings [10,20,22,24,26-28]. For example, in a survey of patients impacted by Hurricane Sandy in 2012, a total of 26.3% of participants reported missing dialysis sessions during the storm, and 8% reported missing 3 or more sessions [26,29]. Similarly, postdisaster analyses have reported an increased risk of emergency department visits, hospitalizations, and short-term mortality among patients receiving hemodialysis exposed to a disaster [27,30,31]. All-cause mortality with hurricane exposure among 187,388 patients undergoing dialysis in the United States from 1997 to 2017 increased by 13% compared to those who were not exposed [31]. Furthermore, vulnerability is compounded by both medical and socioeconomic factors, particularly given that kidney disease disproportionately affects racial and ethnic minority groups and those experiencing structural disadvantages across several other social determinants of health [32-39].

The Canadian Disaster Database reported 424 climate-, conflict-, and technology-related disasters from 2000 to 2020 that have resulted in 1741 deaths and 12,792 injuries, displaced 603,549 individuals, and cost more than CAD $19 billion (US $14 billion) [40]. Anecdotal experiences and reports in the lay press suggest that the kidney care community in Canada has faced numerous disasters and emergencies over the past 2 decades [41-45]. For example, in 2023, many remotely located Indigenous people who received dialysis in the satellite facilities had to be evacuated due to the wildfires. In 2021, torrential rain and winds impacted the care of 72 patients, who needed to be evacuated and repatriated to other dialysis units [45]. While, in the United States, dialysis facilities are mandated by federal regulations to have a disaster plan [16], in Canada, there is no such national initiative in disaster preparedness, response, and recovery in kidney care, although some regional isolated efforts exist. We have highlighted the need to champion disaster and emergency risk reduction and management (DERRM) in kidney care in Canada and underscored the importance of a unified national approach to strengthening system resilience [9]. However, advancing such an approach first requires a clearer understanding of existing systems.

### Framework

#### Overview

To better contextualize this, we first developed a framework in DERRM following a scoping review and a content analysis of contemporary literature addressing disaster management in kidney care across the domains of disaster preparedness, response, and recovery [46]. This work included guidelines, recommendations, commentaries, and review articles. Of the 3381 titles and abstracts screened, 85 (2.5%) articles were selected for full-text review, and ultimately, 45 (1.3%) articles were included in our analysis. Notably, none emerged from Canada. The details of the road map have been published, and brief details are mentioned below.

#### The ABC4S of Disaster Preparedness

**Figure 1.**
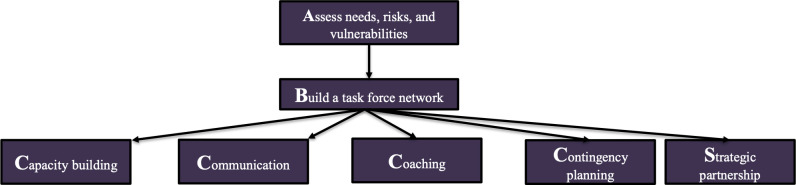
The ABC4S approach to disaster preparedness that we previously developed, which serves as a framework for this vulnerability and adaptation assessment.

This entails a chronological set of processes as follows: assess needs, risks, and vulnerabilities (regional risks and patients at risk); build a task force network; capacity building (tangible resources, intangible resources, monetary considerations, and transportation); communication (network and protocol, patients’ medical and dialysis information, contact information of all stakeholders, inclusive approach, and reliable medium); coaching (patients, caregivers, health care personnel, and reinforce and repeat coaching); contingency planning (surge capacity, rationing care, and resource distribution); and strategic partnerships (Figure 1) [46].

#### The DIAL Disaster Response

On the basis of the existing evidence, we proposed the following circular series of steps: damage and scope assessment; initiate action plan (choose the plan, apply preparedness tenets, and implications for receiving facilities); appraise the action plan regularly (reassess, maintain ethical standards, and address psychosocial needs); and liaise, engage, and update [46].

#### The ARC to Recovery

Although we were guided by limited evidence, we proposed the following steps to create a learning health system: assess damage; return to the (new) norm; and collect data to evaluate, improve, and share [46].

### Research Goals

In the Canadian setting, a better understanding of the experiences, resiliency, and vulnerabilities of existing systems is needed to strengthen preparedness efforts and other risk reduction strategies and build “resilient health systems capable of anticipating, responding to, coping with, recovering from, and adapting to climate-related shocks and stress” [7]. Moreover, a better understanding of system weaknesses, practice gaps, and response capacities can help develop adaptation priority actions that can mitigate risks to patients with kidney diseases. The goal of this research is to conduct a vulnerability and adaptation assessment (VAA) of Canadian provincial health systems and individual kidney care networks (KCNs) with respect to natural and human-caused disasters and emergencies. Our research aims are to map existing KCNs across Canada, contextualize the current and future vulnerability of health systems and patients to disasters and emergencies, and identify and prioritize adaptation measures in partnership with key interest holders. Our overarching goal is to strengthen system preparedness and resilience.

## Methods

We are conducting a bottom-up VAA that follows a qualitative descriptive approach and uses the DERRM framework that we have previously developed (Figure 1). We are using the COREQ (Consolidated Criteria for Reporting Qualitative Research) to guide the conduct and reporting of this work [47].

### Approach

A VAA is a key tool in identifying critical health risks, vulnerable populations, capacities, and weaknesses in the involved systems and the interventions needed to mitigate, manage, and respond to arising risks [7,48,49]. The World Health Organization suggests that VAAs are a foundational step in preparing health systems for hazards. Such an exercise is essential to assess the potential impacts of climate- and human-caused hazards on health systems, profiling the impacted populations, whether patients or health care personnel, and the capacity of health institutions and systems to address arising situations [50]. A VAA explores the components of a health system to assess potential areas of vulnerability to disasters, where capacities stand and/or fail if future risks arise, and what adaptive measures can be taken to achieve a more resilient health system [48]. A bottom-up approach to VAA focuses on the context instead of the outcome of a hazard. It starts with the stakeholders impacted and examines the elements of the involved health systems [51]. Rather than a top-down approach focusing on a particular hazard and estimating its future impact, we used the bottom-up approach to explore the underlying contexts and elements of a system that potentially develop vulnerabilities and limit DERRM [51,52]. Moreover, instead of using model-generated climate data, this approach relies on participatory input from different stakeholder sources to understand disaster management directly from the perspective of those who have experienced or may experience it [52].

### Approach

Often used in nursing and health research, a qualitative descriptive approach is important for gaining insights into underexplored research areas [53]. It is the method of choice when straight descriptions of phenomena are desired and for understanding user experiences of processes within health care systems [54]. Moreover, a qualitative descriptive approach recognizes the subjective nature of research questions in health research and explores the answers through the different perspectives of participants’ experiences [53]. In practice, this translates into understanding important clinical issues within their unique contexts. This approach also benefits from a flexible inductive process to pursue deeper descriptive knowledge, contributing to change and quality improvement in the practice setting without the need to transform data into a theoretical or conceptual reflection [55,56]. Overall, we are adopting a qualitative descriptive design with the intention of obtaining straightforward descriptions of experiences and perceptions in an area with little prior investigation [53,57].

### Data Collection

#### Overview

**Figure 2.**
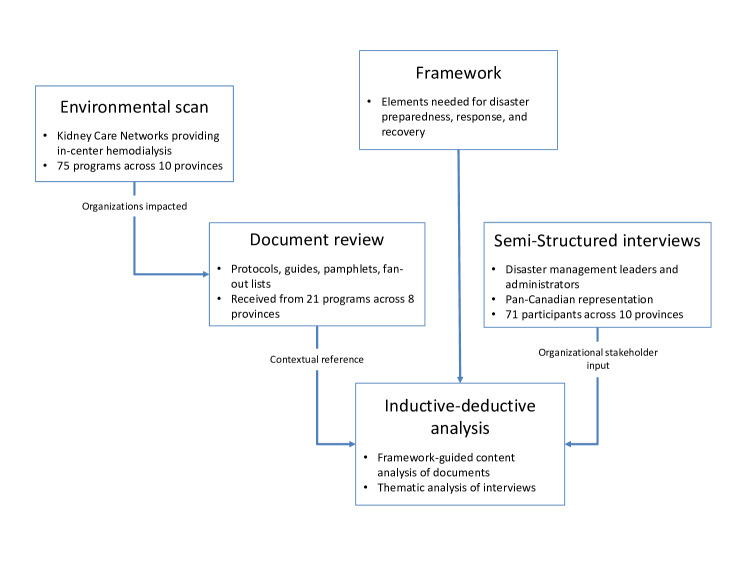
Approach to data collection and analysis in this vulnerability and adaptation assessment research plan of Canadian kidney care networks.

An approach to our data collection is presented in Figure 2 and summarized below.

#### Environmental Scan

In Canada, health care is federally mandated but delivered at the provincial and territorial level across 10 provinces and 3 territories. To establish a clear picture of the kidney care landscape, we began by using data from the Canadian Organ Replacement Register to generate a preliminary map of existing KCNs and dialysis facilities (adult, pediatric, those that are located in cities, and satellite units), home dialysis programs, and transplant programs embedded within each network [58]. Publicly available data from the Canadian Organ Replacement Register provided a foundational national overview; however, as our data collection advanced, additional information from provincial programs, our team, and participants allowed us to refine and update this network structure. The final list of 75 KCNs reflecting these iterative updates and focusing only on adult in-center hemodialysis programs is presented in Table 1. Similar lists will be generated for pediatric, home dialysis, and transplant programs.

**Table 1.** A list of Canadian kidney care networks.

Province, provincial network, and health region	Dialysis network and jurisdiction
British Columbia	
BC Renal	
Fraser Health	Abbotsford Regional Hospital and Cancer Centre Royal Columbian Hospital Surrey Memorial Hospital
Interior Health	Royal Inland Hospital Kelowna General Hospital Penticton Regional Hospital Kootenay Boundary Regional Hospital
Island Health	Nanaimo Regional General Hospital Royal Jubilee Hospital
Northern Health	University Hospital of Northern British Columbia
Vancouver Coastal Health and Providence Health Care	Vancouver General Hospital St. Paul’s Hospital
Alberta	
Alberta Kidney Care – North	University of Alberta Hospital
Alberta Kidney Care – South	University of Calgary
Saskatchewan	
Regina	Regina General Hospital
Saskatoon	St. Paul’s Hospital
Manitoba	
Prairie Mountain Health	Brandon Regional Health Centre
Winnipeg Regional Health Authority	Seven Oaks General Hospital
Shared Health, Northern Health, Interlake-Eastern Regional Health Authority, Prairie Mountain Health, and Southern Health	Health Sciences Centre
Ontario	
Ontario Renal Network	
Dialysis Management Clinics Inc	—^a^
Erie St. Clair	Windsor Regional Hospital
South West	London Health Sciences Centre
Waterloo-Wellington	Grand River Hospital
Hamilton Niagara Haldimand Brant	Niagara Health St. Joseph’s Healthcare Hamilton
Central West	William Osler Health System
Mississauga Halton	Halton Healthcare Trillium Health Partners
Toronto Central	St. Joseph’s Health Centre St. Michael’s Hospital Sunnybrook Health Sciences Centre University Health Network
Central	Humber River Hospital Mackenzie Health
Central East	Lakeridge Health Peterborough Regional Health Centre Scarborough Health Network
South East	Kingston Health Sciences Centre
Champlain	Renfrew Victoria Hospital The Ottawa Hospital
North Simcoe Muskoka	Orillia Soldiers’ Memorial Hospital Royal Victoria Regional Health Centre
North East	Health Sciences North and Horizon Santé-Nord North Bay Regional Health Centre Sault Area Hospital Timmins and District Hospital
North West	Thunder Bay Regional Health Sciences Centre
Quebec	
CISSS^b^ du Bas-Saint-Laurent and CISSS de la Gaspésie	Hôpital régional de Rimouski
CIUSSS^c^ Saguenay–Lac-St-Jean	Hôpital de Chicoutimi
CHU^d^ de Québec–Université Laval, CIUSSS de la Capitale-Nationale, CISSS de la Côte-Nord, and CISSS des Îles	Pavillon L’Hôtel-Dieu de Québec
CIUSSS Mauricie-et-du-Centre-du-Québec	CHAUR^e^ Trois-Rivières
CIUSSS de l’Estrie – CHUS^f^	CHU de Sherbrooke – Hôpital Fleurimont
MUHC^g^ and Conseil cri de la santé et des services sociaux de la Baie James	MUHC
CIUSSS de l’Ouest-de-l’Île-de-Montréal	Centre hospitalier de St. Mary
CIUSSS du Centre-Ouest-de-l’Île-de-Montréal	Hôpital général juif Sir Mortimer B. Davis
CIUSSS de l’Est-de-l’Île-de-Montréal	Hôpital Maisonneuve-Rosemont
CIUSSS du Nord-de-l’Île-de-Montréal Clinique Bois-de-Boulogne	Hôpital du Sacré-Cœur de Montréal
CIUSSS du Centre-Sud-de-l’Île-de-Montréal	Hôpital de Verdun
CHUM^h^	CHUM
CISSS de l’Outaouais	Hôpital de Hull
CISSS de Chaudière-Appalaches	Hôtel-Dieu de Lévis
CISSS de Laval	Hôpital de la Cité-de-la-Santé
CISSS de Lanaudière	Centre hospitalier régional de Lanaudière – Hôpital de Joliette
CISSS des Laurentides and CISSS de l’Abitibi-Témiscamingue	Hôpital régional de Saint-Jérôme
CISSS de la Montérégie-Centre	Hôpital Charles-LeMoyne
CISSS de la Montérégie-Est	Hôpital Honoré-Mercier
CISSS de la Montérégie-Ouest	Hôpital Anna-Laberge
Newfoundland	
Newfoundland and Labrador Health Services	
Eastern, Western, Central, and Labrador-Grenfell Health	Health Sciences Center Hemodialysis Unit Corner Brook Hemodialysis Unit Grand Falls-Windsor Hemodialysis Unit
New Brunswick	
Horizon Health Network	Saint John Regional Hospital
Vitalité Health Network	Dr. Georges-L.-Dumont University Hospital Centre
Prince Edward Island	
PEI Renal Program	Queen Elizabeth Hospital
Nova Scotia	
Eastern, Western, Northern, and Central Health	Queen Elizabeth II Health Sciences Centre Cape Breton Regional Hospital

a Dialysis Management Clinics Inc includes multiple out-of-hospital dialysis units across the province of Ontario and does not fall under a particular dialysis network or jurisdiction.

b CISSS: Les centres intégrés de santé et de services sociaux.

c CIUSSS: Les centres intégrés universitaires de santé et de services sociaux.

d CHU: Centre Hospitalier Universitaire.

e CHAUR: Centre hospitalier affilié universitaire régional.

f CHUS: Centre hospitalier universitaire de Sherbrooke.

g MUHC: McGill University Health Centre.

h CHUM: Centre hospitalier de l’Université de Montreal.

#### Document Review

Once a preliminary list of dialysis networks was generated, we solicited documents to better understand existing practices and policies related to disaster and emergency management. This allows for an examination of different standing policies, directives, and resources on disaster management in each program and will serve as a complementary resource during data analysis [59]. Documents for review were obtained prior to, after, or in conjunction with interviews and included any policy, guideline, directive, resource, or presentation pertaining to disaster management within each dialysis network. Documents nonspecific to these networks, such as hospital-wide emergency protocols, were ineligible. Of the 55 KCNs that have responded thus far, we have procured documents from 21 (38.2%) networks, with 39 (70.9%) confirming they have no formal kidney-specific protocols in place. A list of these documents is provided in Table 2.

**Table 2.** List of documents solicited that will be analyzed during the vulnerability and adaptation assessment.

Province and document title	Health region or dialysis network
British Columbia	
Emergency Management Plan	BC Renal
Renal Department Disaster Response Initiation Chain of Command and Events	BC Renal
Are You Ready: Emergency Preparedness Information for Dialysis Patients	BC Renal
Emergency Preparedness Wallet Card	BC Renal
Emergency Preparedness Month: Planning for disaster - Patient flyer	BC Renal
Emergency Kit Essentials	BC Renal
Alberta	
Pandemic Response Plan – Alberta Kidney Care	Alberta Kidney Care – North
Emergency Preparedness and Response – Hemodialysis	Alberta Kidney Care – South
Emergency Meal Plan for People on Hemodialysis	Alberta Kidney Care – South
Bad Weather Patient Information Package^a^	Alberta Kidney Care – South
Saskatchewan	
Ready or not: Emergency Preparedness Information Booklet for Saskatchewan Dialysis Patients	Regina Kidney Health
Manitoba	
Manitoba Renal Program - Disaster Management Manual	Winnipeg Regional
Ontario	
Emergency Preparedness Plan	Dialysis management clinics
Planning Resource Checklist	Dialysis management clinics
Clinical Services Listing	Dialysis management clinics
Priority Services Checklist	Dialysis management clinics
CKD Surge Demand and Capabilities Checklist	Dialysis management clinics
Critical Infrastructure Checklist	Dialysis management clinics
Emergency Renal Product Supply Checklist	Dialysis management clinics
Dialysis Capital Equipment Inventory	Dialysis management clinics
CKD Hazard Identification and Risk Assessment Workbook	Dialysis management clinics
CKD Hazard Identification and Risk Assessment Instructions for Use	Dialysis management clinics
Emergency Contact Information List	Dialysis management clinics
Staff Planning: Roles and Responsibility	Dialysis management clinics
Staff Planning: Operational and Emergency Full-Time Equivalent Requirements	Dialysis management clinics
Annual Training and Education Checklist	Dialysis management clinics
The Final Checklist	Dialysis management clinics
Emergency Management Planning Guide 2015	Ontario Renal Network
Nephrology Emergency Response Plan	Health Sciences North
Renal Program Leadership Emergency Management Guide	London Health Sciences Centre
Emergency Preparedness Information for Peterborough Regional Renal Program Hemodialysis Patients	Peterborough Regional Health Centre
Emergency Preparedness Information for Peterborough Regional Renal Program Multi-Care Kidney Clinic Patients	Peterborough Regional Health Centre
Emergency Preparedness Information for Peterborough Regional Renal Program Peritoneal Dialysis Patients	Peterborough Regional Health Centre
Peterborough Regional Renal Program Hazardous Identification and Risk Assessment - Emergency Management Plan 2020	Peterborough Regional Health Centre
Regional Renal Program - Emergency Management Plan	Peterborough Regional Health Centre
Algoma Regional Renal Program - Emergency Management Plan	Sault Area Hospital
Dialysis Patients: Being Prepared for an Emergency	Sault Area Hospital
Kidney Care Contingency Plan	St. Joseph’s Health Centre
Emergency Management Plan - Regional Kidney Care Program Simcoe Muskoka	Orillia Soldiers’ Memorial Hospital
Quebec	
Plan d’urgence - Plan particulier d’intervention (PPI)	Centre hospitalier universitaire de Québec
Procédure interne de communication entre les coordonnateurs d’activités de soins et les AIC du Centre de services ambulatoires de dialyse de Gaspé et des sites résiduels de Notre-Dame et Saint-Luc.	Centre hospitalier de l’Université de Montréal
Plan des mesures d’urgence de la dialyse - Eau Contaminée	Centre hospitalier de l’Université de Montréal
Plan des mesures d’urgence de la dialyse - Panne de Courant	Centre hospitalier de l’Université de Montréal
Plan des mesures d’urgence de la dialyse - Panne d’alimentation en eau ou du traitement d’eau	Centre hospitalier de l’Université de Montréal
Plan des mesures d’urgence de la dialyse - Évacuation	Centre hospitalier de l’Université de Montréal
Mesure d’urgence pour patients - Gaspé	Centre hospitalier de l’Université de Montréal
Mesure d’urgence Mesure d’urgence – Fournitures essentielles d’accès vasculaire^a^	Centre hospitalier de l’Université de Montréal
Plan d’urgence - Suppléance Rénale	CIUSSS^b^ de l’Est-de-l’Île-de-Montréal
Liste des machines et du nombre de patients dialysés	CIUSSS du Nord-de-l’Île-de-Montréal
Dialysis Patients: Being Prepared for an Emergency	MUHC^c^
Procedure for Evacuation of Dialysis Patients During Hemodialysis	MUHC
Plan De Contingence Hémodialyse	CIUSSS de l’Estrie – CHUS^d^ Sherbrooke
Plan Contingence Plan Répartition Quotas 4 Patients/Inf.	CIUSSS de l’Estrie – CHUS Sherbrooke
Prince Edward Island	
Renal Program Storm Planning Quick Reference Checklist	PEI Renal Network
Provincial Renal Program Storm Plan	PEI Renal Network
Storm Planning Notification	PEI Renal Network
Queen Elizabeth Hospital Hemodialysis Storm Planning	PEI Renal Network
Nova Scotia	
Emergency Backup Hemodialysis Infrastructure Table	Nova Scotia Health Authority
Emergency Patient Status/Transfer Requirements Summary Form	Nova Scotia Health Authority
Renal Program Business Continuity Status Checklist	Nova Scotia Health Authority
NSHA Renal Program Disaster Evacuation Response	Nova Scotia Health Authority
NSHA Renal Program Emergency Management Action Plan - allocation table	Nova Scotia Health Authority
NSHA Renal Program Essential Services	Nova Scotia Health Authority
NSHA Renal Program Extended Power Outage	Nova Scotia Health Authority
NSHA Renal Program Severe Weather Procedure	Nova Scotia Health Authority
NSHA Renal Program Short Term Power Outage Procedure	Nova Scotia Health Authority
NSHA Renal Program Short Term Water Disruption Procedure	Nova Scotia Health Authority

a Title created based on document content.

b CIUSSS: Les centres intégrés universitaires de santé et de services sociaux.

c MUHC: McGill University Health Centre.

d CHUS: Centre hospitalier universitaire de Sherbrooke.

#### Semistructured Interviews

Finally, we are conducting semistructured interviews with health care professionals (clinicians, administrators, technicians, nurses, and other allied health professionals) exploring the vulnerabilities and service gaps of existing systems, identifying strengths, and discussing potential adaptations to increase the resilience of kidney care in the face of disasters. The guide explores participants’ experiences with disasters and disaster management within their networks or their specific dialysis unit. We investigate participants’ roles, and depending on the extent of their experience with a disaster, participants are prompted to describe lived experiences and their perspectives on the state of disaster preparedness and response at their units or networks. Questions explore different components of the health system and disaster vulnerability, current capacities, associated challenges, and potential adaptations. For example, topics include the comprehensiveness and maintenance of current protocols at the unit; leadership and coordination; aspects of patient preparedness and vulnerability; personnel training and capacities; and different infrastructure systems such as physical, financial, and communication.

The second part of the interview solicits feedback on the developed DERRM framework as a potential adaptation at their unit. All 3 components of the framework are shown to participants, and feedback is collected on its different elements, comprehensiveness, feasibility, and applicability at their units. A complete interview guide is provided in Multimedia Appendix 1. Interviews follow an iterative approach whereby issues or ideas identified by participants are discussed with subsequent participants to enable further definition and refinement of themes [60]. Interviews are conducted remotely through Zoom (Zoom Video Communications) in English or French by trained qualitative researchers. All interviews are being digitally recorded and transcribed.

#### Eligibility and Recruitment

Our eligibility criteria are participants who have a key leadership, administrative, or operational role in a KCN. Relying on the comprehensive list of 75 identified KCNs following the environmental scan, recruitment will focus on the main units within each network as managerial and administrative functions are set by them and followed accordingly by other units within the network. We use purposive criterion sampling to identify these individuals through Google searches, phone calls to kidney units, and the assistance of our collaborators. We focus on identifying a minimum of 1 participant per KCN. Considering varying geographical contexts and different levels of disaster exposure, and to allow for more nuanced experiences, some participants in nonmanagerial roles or from satellite KCNs are also being invited using a snowballing method [61,62]. We will pursue recruitment at each of the 75 KCNs and plan to invite at least 2 contacts within each network a maximum of 3 times. This will ensure accuracy, comprehensiveness, and integrity as all KCNs will be offered an opportunity to participate [63]. If a main unit is nonresponsive, we will move on to other units within that network prior to labeling it as a nonresponding KCN. Recruitment will continue beyond the point of data saturation to pursue pan-Canadian representation.

### Data Analysis

#### Overview

We will use an inductive-deductive approach whereby the DERRM framework guides our data interpretation while also allowing space for emerging themes. Although inductive analysis is more common in qualitative research [64], a deductive framework-guided approach also provides a conceptual map to contextually incorporate data and interpret them in connection with existing literature and evidence [64-67]. This combined analysis approach creates space for different nuances—such as interprovincial differences, rural vs urban settings, and different stakeholder inputs—while maintaining conceptual integrity. As a result, a comprehensive assessment of Canadian KCN vulnerabilities and the needed adaptations can integrate current risk considerations into existing decision-making and health system components [51]. We will use the following stepwise approach.

#### Document Review

We will conduct a deductive framework-guided content analysis. Using the DERRM framework categories, we will develop a coding scheme matrix of the different elements and subelements of disaster management. Document passages will be organized into predefined matrix categories and coded accordingly, with attention paid to data that fit more than one category. The documents’ contents will be coded line by line with the DERRM framework elements as a guide and cross-referenced to populate the matrix with the relevant data. Once the matrix is filled, we will synthesize which categories were more strongly, weakly, or not represented across the documents. This systematic approach will help understand the existing components of disaster management at Canadian KCNs, keeping in mind that the presence of fragmented directives or policies or the absence of any directives is a similarly significant finding. We will also note substantial contradictions and remain open to any categories and subcategories that might emerge [68]. Supporting data, such as rural vs urban location of the unit, size of the unit, size of the network, and province, will also be considered during the analysis.

#### Semistructured Interviews

The interviews will be analyzed using inductive and deductive thematic analysis. Thematic analysis entails identifying and analyzing patterns of meaning and mapping regularities and variations across different accounts [69-72]. An inductive and deductive approach emerges by using the existing DERRM framework to fit our data into an a priori concept [73,74] while also allowing for any new themes and codes to emerge through a close reading of the data [72,75,76]. Interview transcripts will be analyzed independently by 2 senior qualitative researchers. NVivo (version 15; Lumivero) will be used to support data management and analysis.

Transcripts will be read and highlighted line by line to preliminarily derive data anchored in the DERRM framework elements and tease out emerging ideas and themes. These initial findings will be organized into codes and subcodes separately by each researcher to form 2 parallel initial coding schemes. The team will meet to discuss and establish whether intercoder reliability is achieved and decide on a first draft of the codebook. We will ensure rigor throughout the research process, drawing on concepts of transparency, credibility, transferability, and reflexivity as indicators of research quality in qualitative research [77,78]. Transparency will be ensured through the maintenance of a research journal documenting all decisions related to sampling and analysis. Reflexivity will be supported through ongoing discussions of emergent codes and themes alongside critical reflection on analytic decisions and the researchers’ potential influence on the process. The final themes will be presented to the collaborators prior to manuscript preparation.

### Ethical Considerations

Ethics approval for this study was obtained from the Research Ethics Board at McGill University Health Centre, Montreal, Quebec (2025-11213). This study is being conducted in accordance with the Tri-Council Policy Statement: Ethical Conduct for Research Involving Humans 2 (2022) [79].

Written or electronic informed consent will be obtained from interview participants via email prior to commencing the interviews. It will briefly explain the study and its purpose, the voluntary nature of participation, the duration of the interview, and that any personally identifiable information will not be collected or will be omitted from the transcripts. Participants will be compensated with a CAD $50 (US $38) gift card following their participation in the interviews.

## Results

Funding for this research was obtained in April 2025. The environmental scan was initiated in November 2024, and the semistructured interviews were initiated in February 2025. Interviews are ongoing, and the analysis has been initiated simultaneously. We have recruited 71 participants from 55 KCNs from March 2025 to December 2025 and are attempting to recruit at least one participant from the remaining 20 KCNs. The first VAA focusing on in-center hemodialysis services is expected to conclude in April 2026. These findings are projected to be written up by May 2026, and manuscripts are expected to be submitted for publication to peer-reviewed journals in the summer of 2026 (tentatively June 2026). Thereafter, similar VAAs will be conducted focusing on other kidney replacement therapies and pediatric programs. Separate analysis and reporting will be considered depending on emerging codes and resulting themes. Key contacts at KCNs across the different provinces who have supported participant identification will be involved in data interpretation and manuscript preparation. Key insights are emerging with respect to differences in the provision of kidney care; a separate analysis reporting these findings will be considered with the input of our collaborators.

## Discussion

### Expected Findings

This study aims to operationalize a DERRM framework to conduct a VAA of KCNs in Canada. This pan-Canadian initiative will identify and address the vulnerability of the kidney health community (ie, health care providers, patients with kidney disease, kidney care units, and provincial renal health authorities), identify approaches to adaptation, facilitate DERRM, and strengthen health system resilience to disasters. We want to identify the existing operational and human vulnerabilities and the associated risks, as well as explore lessons learned from previous experiences, to develop needed adaptations. Emerging data suggest that this varies widely between programs and provinces. As mentioned above, only 38.2% (21/55) of the responding programs have written documents specifying policies on disaster management. Input gathered has covered managerial and operational aspects, ensuring relevance to applicable policy and lived practices and experiences [7]. The snowball recruitment approach enabled us to reach individuals who are often difficult to engage, including those supporting rural communities and working in direct, frontline roles with patients [46]. These participants contributed valuable experiences in managing disasters through working with different dialysis modalities, holding tenure in their institutions, working with remote communities, and being exposed to a multitude of hazards. All participants interviewed thus far have offered differing perspectives on disaster management in different Canadian contexts. Document review will serve as complementary data collection and as a means of triangulation with interview data [59]. By examining both existing directives and lived experiences and the expertise of health care professionals (clinicians, administrators, technicians, nurses, and other allied health professionals), this approach provides a comprehensive assessment of DERRM at the individual, provincial, and health system levels while directly advancing our research aims.

This will be the first VAA of kidney care facilities across Canada. In addition to investigating vulnerabilities and adaptations at the KCN level, analysis will highlight provincial and regional variations based on prior exposure to disasters and perceived current and future risks. Future research will explore patient and caregiver perspectives and adaptation recommendations to generate actionable goals to guide relevant interest holders in prioritizing and planning responses to current and future health threats. Furthermore, as per the World Health Organization, VAA can serve as a baseline for monitoring changes in risk and evaluating the effectiveness of adaptation measures over time.

### Limitations

The following limitations may be present in our analysis, and we are undertaking steps to address some of them. First, despite extensive consultations with collaborators and ongoing verification during interviews, we may not have fully captured all existing KCNs in Canada. Although we attempted to engage every KCN to achieve pan-Canadian representation, several regions could not be reached to validate their inclusion, and we are continuing efforts to engage with them. During our ongoing data collection, significant differences are emerging regarding the vulnerabilities of patients on various kidney replacement therapies. We are attempting to recruit participants who will represent the full scale of therapies across adult and pediatric populations.

Regarding our methods, our recruitment approach may be susceptible to nonresponse bias as only those with a personal interest in the research topic may have agreed to participate. Additionally, those who perceived their KCN as insufficiently prepared may have opted not to participate, potentially limiting the range of perspectives captured. This may potentially skew insights into system vulnerabilities and the applicability of potential adaptations. However, we are undertaking several measures to increase our outreach efforts to ensure that all perspectives are represented. Finally, although the document review serves as a contextual reference for existing policies on disaster management at KCNs complementary to interview data, we acknowledge that it may not be exhaustive as documents are shared through participants and dependent on their knowledge of their own unit’s directives or lack thereof.

The target population focused on the main dialysis center within each KCN, and we only recruited participants from satellite units through snowballing when recommended. We acknowledge that other units within a network can also offer valuable, nuanced input; however, considering that administrative functions and managerial functions are localized to main units, we believe that this level of representation is sufficient. In parallel, the study examines organizational-level vulnerabilities and adaptations without addressing individual-level risk factors, including patient experience with different kidney replacement therapy modalities. Patient perspectives will be pursued in the following stages of the study.

### Conclusions

Patients with kidney diseases are uniquely vulnerable to the impacts of a hazard. Delay in the provision of dialysis or medications can be a life-threatening or organ-threatening event for these patients. This VAA, guided by a framework we previously developed within the DERRM model and informed by an extensive literature review and experiential knowledge, represents the first initiative of its kind in kidney care. The findings will provide a critical foundation for understanding vulnerability, supporting strategic planning, and guiding adaptation measures that can strengthen health system resilience. Ultimately, this work will help protect patients with kidney diseases and the broader kidney community from the escalating risks of evolving hazards within Canada and may serve as a model for similar efforts elsewhere.

## Supplementary material

10.2196/90059 Multimedia Appendix 1 Semistructured interview guide for health care professionals.

10.2196/90059 Peer Review Report 1 Peer review report by the Clinical Investigation C: Digestive, Endocrine and Excretory Systems (CIC) Review Committee, Canadian Institutes of Health Research (CIHR).

## Abbreviations:

COREQ: Consolidated Criteria for Reporting Qualitative Research
DERRM: disaster and emergency risk reduction and management
KCN: kidney care network
VAA: vulnerability and adaptation assessment
