# Supplementary material for: All-Hazards Vulnerability and Adaptation Assessment of Canadian Kidney Care Systems: Protocol for a Qualitative Study
Source: JMIR Res Protoc. 2026 Apr 23;15:e90059. doi: 10.2196/90059 (PMC13105280; doi:10.2196/90059)
Supplement: Multimedia Appendix 1 [file resprot-v15-e90059-s001.docx]

Appendix 1:

Interview guide: Healthcare providers

A brief about the study

[This study is being conducted by the McGill University Health Centre (MUHC) and is funded by a grant from the Canadian Institute of Health Research. Dr. Shaifali Sandal is the lead researcher for this study; I am a researcher working on the project. This study examines the vulnerabilities of kidney care facilities to disasters and operationalizes a disaster risk reduction and management approach, published in Clinical Journal of the American Society of Nephrology. We have identified you as someone in a leadership position in a kidney care facility or as someone responsible for leading disaster management]

Explain the process of consent-

- 1. audio-recorded and will be transcribed without any personal identifiers.
  2. Anything discussed during the interview will be anonymized
  3. You have right to withdraw comments from study
  4. You have the right to terminate the interview at any time prior to the closure of the study, without any explanation and without penalty and have your transcript removed from the study record. Once the study is closed and results have been disseminated, you will no longer be able to withdraw from the study.
  5. Data (including direct quotes) from the interview may be included in publications and in scholarly presentations, but no personal identifying data will be included.

**Any questions before we start?**

**Ok, I will now start recording**

**[start recording]**

**PART-1 for all respondents**

1. Years of practice
2. Role
3. Number of units under network
4. Representation level- (provincial, local, …)
5. Adult or Pediatric
6. Number of patients
7. Treatment modality
8. Guidelines in place
9. **Background Information**

- Can you please describe your role in the dialysis unit?

[ Probe on Managerial/patient care role]

**[ How does the network engage in planning, responding and recovery of disasters? Can you please explain]**

- Does your unit cater to a vulnerable population? Can you please describe these groups?
- Does your centre also cater to home dialysis?
- Does your role involve planning for disasters? Can you please explain

1. **Disaster Information**

- What would you describe as a disaster?
- What disasters are common in your region?
- Have you witnessed/experienced or helped manage a disaster as a HCP?

[ If the answer is yes then proceed to **section C** and if no then proceed to **section D]**

- Can you describe the preparations and protocols that are in place to manage a disaster like X at the centre?

1. **Experience in managing a disaster**
2. Can you describe the extent of the disaster and how it was managed?

[Probe on immediate, intermediate and long-term effects & impacts on patients and facility]

1. What were the key challenges during the disaster?

[Probe- lack of a guideline/planning, coordination between agencies, lack financial and personnel support, transportation, early evacuation, sharing of medical record

1. Would you say that not having a formal guide complicates things?
2. Can you enumerate the facilitators for leading a good disaster response

[Probe- planning, coordination, resources]

1. What would you do differently if you were to plan for the disaster or respond to it?
2. Would you say that your patients are well prepared for the next disaster?

- what can be done to improve that?

1. What have been the key learnings in the process of managing a disaster?
2. What about staff? Are there any protocols/provisions/considerations aimed at protecting staff or organizing their work during disasters?
3. **Disaster readiness for centres without disaster occurrence or indirectly affected**
4. What would you like to share about your centre’s efforts towards a disaster readiness plan?

[Probe- a guideline, contingency planning, preparedness at the level of patient and healthcare staff]

1. What factors can enable better preparedness for disaster?

[Probe- protocol, guidance, team]

1. Can you describe the challenges of preparing for a disaster?

[Probe- resources (human and financial)]

1. How can we overcome these challenges?

**PART-2 for respondents with managerial positions and above/ OR who have faced a disaster in the past**

**As a part of our research, we would like to show you the disaster management roadmap we have generated following a review of literature on DRRM that has been recently published in the journal XX. The framework has three parts- ABC’s focuses on preparedness, the DIAL (damage assessment, initiate action plan, assess and reassess, and liaise with strategic partners) focuses on the response and ARC ( to recovery. We will go over its elements step by step while also discussing your feedback and your real-life experience with the disaster.**

**[Show the ABC framework]**

1. What would you say were the most effective elements in addressing disasters at your centre? literature indicates that it’s preparation and we have put together these key elements on preparation, what are your thoughts?

- [probe on each element of the framework 1) Capacity building - tangible and intangible resources, along with transportation, and monetary considerations. 2)Communication- medium, with stakeholders and patients, telemedicine. 3)Coaching- training available for staff & patients. 4) Contingency planning- surge capacity, resource distribution. 5) strategic partnership- Government, local/provincial agencies, industry partner
- What are your thoughts on creating a task force with a disaster lead at each site? How can it be done? What would be key considerations for initiating a task force? What may be required as memorandum between partners.

[Probe- What level would be more effective/efficient regional/provincial]

- What are your thoughts on its feasibility at your centre?

1. With respect to **response**, we propose a DIAL response- having elements of damage assessment, initiate action plan, assess and reassess, and liaising with strategic partners

**[ Show DIAL framework]**

- What are your thoughts on the framework? Does it cater to all elements?

Would you like to add anything?

[Probe- any difference in hemodialysis/ home dialysis].

- How frequently do you think this response needs to be updated?
- What are your thoughts on ethical standards while creating a response?
- How has your unit dealt with the personal effects of the disaster?

[Probe on the mental health aspect-support available to staff and patients]

1. Moving on to the recovery, we found limited literature to guide us. We propose this roadmap to recovery, we call it ARC to recovery

**[ Show recovery framework]**

- What are the key considerations for going back to normal after a disaster?
- What can be the resources or assistance that can enable a swift recovery

[Probe- for the institution, patient]

- How can we improve the recovery?

[Probe on data and its reporting and usage]
