# Supplementary material for: All-Hazards Vulnerability and Adaptation Assessment of Canadian Kidney Care Systems: Protocol for a Qualitative Study
Source: JMIR Res Protoc. 2026 Apr 23;15:e90059. doi: 10.2196/90059 (PMC13105280; doi:10.2196/90059)
Supplement: Peer Review Report 1 [file resprot-v15-e90059-s002.pdf]

CIHR IRSC | Canadian Institutes of Health Research / Instituts de recherche en santé du Canada

15 January/Janvier 2025

Notice of Recommendation / Avis de recommandation

Project Grant Program / Programme de subventions Projet

**Nominated Principal Applicant / Candidate principale désignée ou Candidat principal désigné :**	Sandal, Shaifali
**Principal Applicants / Candidats principaux :**	
**Co-Applicants / Cocandidats :**	Ethier, I; Finkle, N; Glavinovic, T; Horowitz, L; Hunt, M; Macrae, JM; Samanta, R; Stigant, CE; Weber, CL; Yohanna, S
**Institution Paid / Établissement payé :**	Research Institute of the McGill University Health Centre / Institut de recherche du Centre universitaire de santé McGill
**Title / Titre :**	Disaster risk reduction and management in kidney care to foster health system resilience
**Application Number / Numéro de la demande :**	526060

Competition information / Information du concours

**Competition / Concours :**	202409PJT
**Applications in competition / Demandes dans le concours :**	2631

Peer Review Committee Recommendation / Recommandation du comité d'évaluation par les pairs

Committee Name / Nom du comité : Clinical Investigation - C: Digestive, Endocrine and Excretory Systems (CIC) / Investigation clinique - C : Systèmes digestif, endocrinien et excrétoire (CIC)

Applications reviewed / Demandes évaluées : 31

Your percent rank zone / Votre intervalle de rangs centiles :

100%-90%	89.99%-75%	**74.99%-50%**	49.99%-25%	24.99%-0%

Final rating / Cote finale : 3.93 (Discussed / Demande ayant fait l'objet d'une discussion)

Recommended term / Durée recommandée : 3 years/ans 0 months/mois

Recommended average annual operating amount / Montant annuel moyen recommandé pour le fonctionnement : $175,964

Is your application a large grant? / Est-ce que votre demande est une grande subvention? No

Additional information / Autres Informations

This document is for information only. An application rated below 3.50 is ineligible for CIHR funding. The final funding decision will be communicated in the Notice of Decision.

Document à titre d'information seulement. Une demande cotée en dessous de 3,50 n'est pas admissible au financement des IRSC. La décision finale relative au financement sera communiquée dans l'Avis de décision.

The ratings in your written reviews are the preliminary ratings from the assigned reviewers of your application. The final rating on this document represents the average of the ratings following committee discussion. As such, preliminary ratings and the final rating may differ. For more information, visit https://cihr-irsc.gc.ca/e/51315.html.

Les cotes figurant dans vos évaluations écrites correspondent aux cotes préliminaires attribuées par les évaluateurs à qui votre demande était assignée. La cote finale figurant sur ce document représente la moyenne des cotes à la suite de la discussion du comité. Ainsi, les cotes préliminaires et la cote finale peuvent différer. Pour de plus amples renseignements à ce sujet, consultez la page https://cihr-irsc.gc.ca/f/51315.html.

**Review Type / Type d'évaluation:**	Reviewer 1 / Évaluateur 1
**Name of Applicant / Nom du chercheur:**	Sandal, Shaifali
**Application No. / Numéro de demande:**	526060
**Agency / Agence:**	CIHR/IRSC
**Competition / Concours:**	Project Grant/Subvention Projet
**Committee / Comité:**	Clinical Investigation - C: Digestive, Endocrine and Excretory Systems/Investigation clinique - C : Systèmes digestif, endocrinien et excrétoire
**Title / Titre:**	Disaster risk reduction and management in kidney care to foster health system resilience

Adjudication Criteria/Critères de sélection

Initial Score/Cote Initiale: 4.6

Top/Bottom Selection/Groupe supérieur/inférieur

- (●) **Top/Groupe supérieur**
- ( ) **Bottom/Groupe inférieur**

**Review Type / Type d'évaluation:**	Reviewer 1 / Évaluateur 1
**Name of Applicant / Nom du chercheur:**	Sandal, Shaifali
**Application No. / Numéro de demande:**	526060
**Agency / Agence:**	CIHR/IRSC
**Competition / Concours:**	Project Grant/Subvention Projet
**Committee / Comité:**	Clinical Investigation - C: Digestive, Endocrine and Excretory Systems/Investigation clinique - C : Systèmes digestif, endocrinien et excrétoire
**Title / Titre:**	Disaster risk reduction and management in kidney care to foster health system resilience

Summary of Application/Résumé de la demande:

Access to essential medical care can be compromised by natural disasters, including extreme weather events, earthquakes and tsunamis, and by social disruptions (such as riots and labour strikes), cyberattacks and armed conflict. These events pose extreme risks to life and health to patients with advanced chronic kidney disease and kidney transplants because of damage or destruction of buildings, equipment and medical records. An effective response to such events requires advance planning, preparation and rehearsal with involvement of health-care systems, professionals, patients and their support networks. To date, unfortunately, there has been little Pan-Canadian discussion and preparation in this respect. These investigators from across the country propose to study the current state of disaster readiness of kidney health programs in Canada. The fragmented nature of emergency management in Canada with devolved responsibilities shared across municipal, provincial and federal authorities without a central coordinating agency such as FEMA in the USA makes it challenging to find a single conduit to the federal government. However, the fragmented nature of Emergency Management in Canada makes an initiative such this more important.

The stated aims of the project are:

1.Conduct an environmental scan of kidney care programs across Canada to understand the existing vulnerabilities and identify strengths and service gaps.

2.Conduct an in-depth exploration of the perspectives of patients, caregivers, and healthcare professionals in kidney care who were directly or indirectly impacted by a disaster.

3.Develop consensus-generated guidelines in disaster risk reduction and management using a Delphi approach. Participants will include a Pan-Canadian representation of varied stakeholders and 4-5 working groups. They will work on different domains of disaster risk reduction and management and develop statements that will be rated during a 1-day consensus conference.

**Review Type / Type d'évaluation:**	Reviewer 1 / Évaluateur 1
**Name of Applicant / Nom du chercheur:**	Sandal, Shaifali
**Application No. / Numéro de demande:**	526060
**Agency / Agence:**	CIHR/IRSC
**Competition / Concours:**	Project Grant/Subvention Projet
**Committee / Comité:**	Clinical Investigation - C: Digestive, Endocrine and Excretory Systems/Investigation clinique - C : Systèmes digestif, endocrinien et excrétoire
**Title / Titre:**	Disaster risk reduction and management in kidney care to foster health system resilience

Strengths and Weaknesses/Forces et faiblesses:

Strengths:

Strong group of researchers with extensive international network connections and a lead investigator who has excellent credentials in nephrology and disaster management.

There is an urgency in obtaining information on disaster readiness from kidney health programs on a national basis in Canada. This will incentivize cooperation from the many groups that will be consulted.

The Delphi process allows for the groups identified to meet, hear each other's viewpoints and develop networks and is ideal for a project such as this.

The reluctance of many provincial health departments across the country to openly share details related to COVID infections, hospitalizations and deaths has shown how difficult it is to obtain health information on a country-wide level from government sources in some provinces. However, the involvement of researchers from academic centres across the country makes it likely this study can succeed in obtaining the necessary information and hopefully encourage the provinces and federal authorities to develop coordinated disaster plans.

Weaknesses:

The fragmented nature of emergency management in Canada with devolved responsibilities shared across municipal, provincial and federal authorities without a central coordinating agency such as FEMA in the USA makes it challenging to find a single conduit to the federal government. However, the fragmented nature of Emergency Management in Canada makes an initiative such this even more important.

The proposal should also specifically include and discuss the risks, prevention and management of cyberattacks on health informatics and healthcare.

It may be challenging to get cooperation and involvement from all the groups identified in the Delphi process.

**Review Type / Type d'évaluation:**	Reviewer 1 / Évaluateur 1
**Name of Applicant / Nom du chercheur:**	Sandal, Shaifali
**Application No. / Numéro de demande:**	526060
**Agency / Agence:**	CIHR/IRSC
**Competition / Concours:**	Project Grant/Subvention Projet
**Committee / Comité:**	Clinical Investigation - C: Digestive, Endocrine and Excretory Systems/Investigation clinique - C : Systèmes digestif, endocrinien et excrétoire
**Title / Titre:**	Disaster risk reduction and management in kidney care to foster health system resilience

Budget Recommendation/Recommandation budgétaire:

The budget is appropriate and reasonable for a project of this scope, duration and importance.

Please indicate your appraisal of the integration of sex as a biological variable as a strength, weakness, or not applicable to the proposal./Prière de sélectionner une option pour donner votre évaluation de l'intégration du sexe comme variable biologique en tant que point fort ou point faible de la proposition, ou en tant qu'élément non applicable à la proposition.

- ○ **Strength/Point fort**
- ○ **Weakness/Point faible**
- ◉ **Not applicable/Non applicable**

Please indicate your appraisal of the integration of gender as a socio-cultural determinant of health as a strength, weakness, or not applicable to the proposal./Prière de sélectionner une option pour donner votre évaluation de l'intégration du genre comme déterminant socioculturel de la santé en tant que point fort ou point faible de la proposition, ou en tant qu'élément non applicable à la proposition.

- ○ **Strength/Point fort**
- ○ **Weakness/Point faible**
- ◉ **Not applicable/Non applicable**

**Review Type / Type d'évaluation:**	Reviewer 1 / Évaluateur 1
**Name of Applicant / Nom du chercheur:**	Sandal, Shaifali
**Application No. / Numéro de demande:**	526060
**Agency / Agence:**	CIHR/IRSC
**Competition / Concours:**	Project Grant/Subvention Projet
**Committee / Comité:**	Clinical Investigation - C: Digestive, Endocrine and Excretory Systems/Investigation clinique - C : Systèmes digestif, endocrinien et excrétoire
**Title / Titre:**	Disaster risk reduction and management in kidney care to foster health system resilience

Sex and/or Gender Considerations/Notions de sexe et/ou de genre:

Considerations of sex and gender are not applicable to the proposal.

**Review Type / Type d'évaluation:**	Reviewer 2 / Évaluateur 2
**Name of Applicant / Nom du chercheur:**	Sandal, Shaifali
**Application No. / Numéro de demande:**	526060
**Agency / Agence:**	CIHR/IRSC
**Competition / Concours:**	Project Grant/Subvention Projet
**Committee / Comité:**	Clinical Investigation - C: Digestive, Endocrine and Excretory Systems/Investigation clinique - C : Systèmes digestif, endocrinien et excrétoire
**Title / Titre:**	Disaster risk reduction and management in kidney care to foster health system resilience

Adjudication Criteria/Critères de sélection

Initial Score/Cote Initiale: 3.9

Top/Bottom Selection/Groupe supérieur/inférieur

- (●) **Top/Groupe supérieur**
- ( ) **Bottom/Groupe inférieur**

**Review Type / Type d'évaluation:**	Reviewer 2 / Évaluateur 2
**Name of Applicant / Nom du chercheur:**	Sandal, Shaifali
**Application No. / Numéro de demande:**	526060
**Agency / Agence:**	CIHR/IRSC
**Competition / Concours:**	Project Grant/Subvention Projet
**Committee / Comité:**	Clinical Investigation - C: Digestive, Endocrine and Excretory Systems/Investigation clinique - C : Systèmes digestif, endocrinien et excrétoire
**Title / Titre:**	Disaster risk reduction and management in kidney care to foster health system resilience

Summary of Application/Résumé de la demande:

Applicants

The NPA is a mid-career, FRQS junior applicant from the McGill University Health Centre (MUHC) who changed from a clinical to a research position in January 2022. The applicant research programs is centered on the assessment and improvement of health systems delivering care to patients with kidney disease. The applicant main clinical and research expertise lies in transplant nephrology, with a more recent uptake in climate change-related disasters and emergencies.

The research productivity is very favorable taking into account specificities of their career pathway and interruptions. More specifically for funding, the NPA received 2 CIHR grants as principal applicant (1 full project grant, 1 early-career patient-oriented research priority funding), 1 CIHR planning and dissemination grant (related to the current application), 2 grants from the Kidney Foundation of Canada as principal applicant and several grants as co-applicants or other institutions. Their publication productivity is good, with a range of first and last authors manuscripts in the recent years, as well as collaborations and co-authorship.

The current project builds on previous work and expertise, more related to kidney transplantation, but also include the NPA expertise in Green nephrology built through the International Society of Nephrology Emerging Leaders program, with collaboration of relevant experts (see below).

Project Description

This is a 3-year proposal aiming to operationalize a framework in disaster risk reduction and management (DRRM) that was recently developed by the applicant team in a previous project. This framework includes a roadmap with 3 sections: 1) the ABC4s of disaster preparedness, 2) the DIAL disaster response, 3) the ARC to recovery.

The current application includes 3 aims:
1) Conduct a vulnerability and adaptation assessment of provincial health system and individual kidney care facilities to natural and human-caused disasters,
2) Conduct an in-depth exploration of the perspective and-or experiences of patients, caregivers and HCP related to priorities, need and system capacities/vulnerabilities during a disaster
3) Develop consensus-based national guidelines in DRRM in kidney care

The proposal uses qualitative methodology for the 3 aims.

Aim 1. Environmental scan of 86 centers throughout Canada (goal is to reach >80%) with an aim to summarize procedure, SOPs, policies, and other documents in place related to DRRM in kidney care centers. A short survey is also planned. The

proposed analytic approach is deductive content analysis.

Aim 2. Patient, caregiver and HDP perspective on DRRM using semi-structured interviews and an interpretive descriptive approach. The applicant did not specify a total target sample size, although minimal number of participants have been identified in different categories including dialysis (n=20), kidney transplant recipients (n=15), caregivers (n=15) and HCPs (n=15). Different targeted proportions of different sous-groups have also been specified.

Aim 3. Developing national guidelines in DRRM using a modified Delphi Method and a consensus-building process with a steering committee and an expert panel of 30 members. A a-day conference is planned to finalize the recommendations.

**Review Type / Type d'évaluation:**	Reviewer 2 / Évaluateur 2
**Name of Applicant / Nom du chercheur:**	Sandal, Shaifali
**Application No. / Numéro de demande:**	526060
**Agency / Agence:**	CIHR/IRSC
**Competition / Concours:**	Project Grant/Subvention Projet
**Committee / Comité:**	Clinical Investigation - C: Digestive, Endocrine and Excretory Systems/Investigation clinique - C : Systèmes digestif, endocrinien et excrétoire
**Title / Titre:**	Disaster risk reduction and management in kidney care to foster health system resilience

Strengths and Weaknesses/Forces et faiblesses:

STRENGTHS

This proposal is well-written, addresses an important question and is led by a productive mid-career applicant.

The study team is diverse in terms of expertise, both on a research perspective basis but also with inclusion of nurses, administrators and knowledge users.

The project is supported by Can-SOLVE CKD, a SPOR network, and the important of patient-partners and their active role in the study is clearly presented.

Sex and Gender is well considered, mostly in Aim 2, with specification of minimal quota to be targeted in participants.

Equity and diversity is a strength of this proposal.

Qualitative methods are clearly presented and justified.

Knowledge translation is well present, and in fact, represent a significant proportion of the third aim. The end-of-grant plan is also highly relevant.

WEAKNESSES

Overall, this proposal does not seem at full maturity as the work representing the basis of the proposal, the DRRM framework roadmap, is still not completed. Several concepts of this preliminary work are underlined in the proposal, but a better understanding of this ongoing work would deepen our understanding of the potential impact of the current proposal.

While this work is important, its scope may need to be redefined, especially the third aim which may be at the interphase between research and knowledge translation.

Aim 1.

More methodological details may be relevant to better understand the planned work in this environmental scan.
The content, format, length, number of question and other details of the ‘short survey’ could also be more explicit.

Aim 2.

This is an important objective; however, some important limitations remain. A cut-off of 10 years has been identified to prevent recall bias, which appears quite long. A shorter time limit such 5 yrs may be more relevant. Use of international collaboration from Australian colleagues as a mitigation strategy is also worrisome as this project is meant to explore the Canadian reality.

The targeted sample size (or an estimation) should be added to this section in addition to the minimal sample in each category. Justifications for the chosen minimum quotas should be added. 30% of participants non-English/non-French speaking appear as a high proportion. Are these meant to be non-native French or English speaking (which is different). A minimal proportion of French speaking should also probably be indicated as well to be more representative of Canadian reality.
Remote communities and satellite units are not synonyms. Several centers have satellite units that would not be considered as remote communities.

There is no indication of regions and centers in which these participants will be recruited. Although it is understood that collaborations and partner organization will contribute to the recruitment, more information are needed to ensure the representatively of this work is warrant.

Aim 3.
Again, more details on the planned modified Delphi method would improve this application.
What are the expected deliverables and how will the expert group ensure these recommendations feasible across different Canadian clinical care settings?

**Review Type / Type d'évaluation:**	Reviewer 2 / Évaluateur 2
**Name of Applicant / Nom du chercheur:**	Sandal, Shaifali
**Application No. / Numéro de demande:**	526060
**Agency / Agence:**	CIHR/IRSC
**Competition / Concours:**	Project Grant/Subvention Projet
**Committee / Comité:**	Clinical Investigation - C: Digestive, Endocrine and Excretory Systems/Investigation clinique - C : Systèmes digestif, endocrinien et excrétoire
**Title / Titre:**	Disaster risk reduction and management in kidney care to foster health system resilience

Budget Recommendation/Recommandation budgétaire:

The budget could probably be significantly reduced without jeopardizing the feasibility of the project.

Please indicate your appraisal of the integration of sex as a biological variable as a strength, weakness, or not applicable to the proposal./Prière de sélectionner une option pour donner votre évaluation de l'intégration du sexe comme variable biologique en tant que point fort ou point faible de la proposition, ou en tant qu'élément non applicable à la proposition.

- (●) **Strength/Point fort**
- ( ) **Weakness/Point faible**
- ( ) **Not applicable/Non applicable**

Please indicate your appraisal of the integration of gender as a socio-cultural determinant of health as a strength, weakness, or not applicable to the proposal./Prière de sélectionner une option pour donner votre évaluation de l'intégration du genre comme déterminant socioculturel de la santé en tant que point fort ou point faible de la proposition, ou en tant qu'élément non applicable à la proposition.

- (●) **Strength/Point fort**
- ( ) **Weakness/Point faible**
- ( ) **Not applicable/Non applicable**

**Review Type / Type d'évaluation:**	Reviewer 2 / Évaluateur 2
**Name of Applicant / Nom du chercheur:**	Sandal, Shaifali
**Application No. / Numéro de demande:**	526060
**Agency / Agence:**	CIHR/IRSC
**Competition / Concours:**	Project Grant/Subvention Projet
**Committee / Comité:**	Clinical Investigation - C: Digestive, Endocrine and Excretory Systems/Investigation clinique - C : Systèmes digestif, endocrinien et excrétoire
**Title / Titre:**	Disaster risk reduction and management in kidney care to foster health system resilience

Sex and/or Gender Considerations/Notions de sexe et/ou de genre:

Sex and Gender is well considered, mostly in Aim 2, with specification of minimal quota to be targeted in participants.

**Review Type / Type d'évaluation:**	Reviewer 3 / Évaluateur 3
**Name of Applicant / Nom du chercheur:**	Sandal, Shaifali
**Application No. / Numéro de demande:**	526060
**Agency / Agence:**	CIHR/IRSC
**Competition / Concours:**	Project Grant/Subvention Projet
**Committee / Comité:**	Clinical Investigation - C: Digestive, Endocrine and Excretory Systems/Investigation clinique - C : Systèmes digestif, endocrinien et excrétoire
**Title / Titre:**	Disaster risk reduction and management in kidney care to foster health system resilience

Adjudication Criteria/Critères de sélection

Initial Score/Cote Initiale: 4.0

Top/Bottom Selection/Groupe supérieur/inférieur

- (●) **Top/Groupe supérieur**
- ( ) **Bottom/Groupe inférieur**

**Review Type / Type d'évaluation:**	Reviewer 3 / Évaluateur 3
**Name of Applicant / Nom du chercheur:**	Sandal, Shaifali
**Application No. / Numéro de demande:**	526060
**Agency / Agence:**	CIHR/IRSC
**Competition / Concours:**	Project Grant/Subvention Projet
**Committee / Comité:**	Clinical Investigation - C: Digestive, Endocrine and Excretory Systems/Investigation clinique - C : Systèmes digestif, endocrinien et excrétoire
**Title / Titre:**	Disaster risk reduction and management in kidney care to foster health system resilience

Summary of Application/Résumé de la demande:

Title: Disaster risk reduction and management in kidney care to foster health system resilience

People with kidney diseases are vulnerable to natural and human-caused disasters, and emergency organizations may under-recognize the urgency of providing therapies such as dialysis and immunosuppressive medications. Background work completed by the research team has included a scoping review and content analysis of the literature, framework development for disaster risk reduction and management (DRRM). This application is focused on operationalizing this framework in the Canadian setting.

The overall goal is “to create resilient health systems in kidney care by building system capacities and decreasing vulnerabilities to future” disasters. This particular proposal has 3 aims:
“Aim 1: Conduct a vulnerability and adaptation assessment of provincial health systems and individual kidney care facilities to natural and human-caused disasters
Aim 2: Conduct an in-depth exploration of the perspectives and/or experiences of:
-Patients and their caregivers to identify their priorities and healthcare needs during a disaster
-HCPs to identify their needs and obtain their opinions on system capacities and vulnerabilities
Aim 3: Develop consensus-based national guidelines in DRRM in kidney care”

Methods used include an environmental scan via document review and survey (Aim 1), semi-structured interviews and thematic analysis (Aim 2), and guideline development using a Delphi approach (Aim 3).

This is a new submission, combining researchers/co-applicants at all levels of experience (early/mid/senior). A knowledge user is also listed as a collaborator.

**Review Type / Type d'évaluation:**	Reviewer 3 / Évaluateur 3
**Name of Applicant / Nom du chercheur:**	Sandal, Shaifali
**Application No. / Numéro de demande:**	526060
**Agency / Agence:**	CIHR/IRSC
**Competition / Concours:**	Project Grant/Subvention Projet
**Committee / Comité:**	Clinical Investigation - C: Digestive, Endocrine and Excretory Systems/Investigation clinique - C : Systèmes digestif, endocrinien et excrétoire
**Title / Titre:**	Disaster risk reduction and management in kidney care to foster health system resilience

Strengths and Weaknesses/Forces et faiblesses:

Strengths:

-The application is very well-written, easy to follow, and organized.

-The nPA appears established as a health services researcher with a clinical focus in nephrology, and appears to have the training/knowledge/experience/expertise required to make study completion feasible. Any delays or impacts on productivity have been well-explained in the Summary of Progress.

-Background is well laid out. Some excellent preliminary work has been done by the nPA and research team with respect to survey/systematic review of perspectives on DRRM.

-Thoughtful presentation of anticipated challenges and mitigation plans for each presented aim.

-KT plan appears achievable

-Excellent letters of support from various stakeholders involved in the project

Weaknesses:

-Regarding the following statement: "In Canada, there is no national initiative in disaster preparedness, response and recovery in kidney care; though some regional, isolated efforts exist." It is the opinion of this reviewer that these "regional, isolated efforts" should be better described in the background of this proposal. The rationale to conduct this study would be strengthened if the application explained what these efforts have been, why they are inadequate, or how they can be improved upon. The argument to fund this study would be more compelling if the present application outlined the current state of DRRM efforts more explicitly, and highlighted how this study proposal can improve on what already exists.

-The application would be strengthened if there were an Indigenous partner included on the study team, in light of the application stating that "in Canada, there is a higher prevalence of kidney diseases and kidney failure necessitating dialysis among Indigenous and other racialized populations…"

-While I commend and wholeheartedly support Aim 2 and agree there is a need for patient/caregiver/HCP perspectives in this body of research, there is a bit of detail lacking for Aim 2: The main proposal does not state who will actually conduct the interviews, although the budget states it will be the post-doctoral fellow. The interaction of the person conducting the interviews could have some influence on the interviewee, and this could be a potential challenge, with mitigating strategies discussed. Additionally, should the sampling attempt to include patients who have experienced a variety of disasters (i.e., natural versus human-caused) in order to obtain more diverse viewpoints, and how can this be guaranteed?

-For aim 3, I would suggest a more explicit description of the composition of the working groups and the domains/aspects of DRRM that will be sub-divided.

-Will there be any KT plan of engagement involving direct communication with the participating survey respondents/kidney care centres from Aim 1?

**Review Type / Type d'évaluation:**	Reviewer 3 / Évaluateur 3
**Name of Applicant / Nom du chercheur:**	Sandal, Shaifali
**Application No. / Numéro de demande:**	526060
**Agency / Agence:**	CIHR/IRSC
**Competition / Concours:**	Project Grant/Subvention Projet
**Committee / Comité:**	Clinical Investigation - C: Digestive, Endocrine and Excretory Systems/Investigation clinique - C : Systèmes digestif, endocrinien et excrétoire
**Title / Titre:**	Disaster risk reduction and management in kidney care to foster health system resilience

Budget Recommendation/Recommandation budgétaire:

Request is for $695,000. This reviewer finds the budget to be somewhat inflated. The methodology being used is 1) Survey/document review; 2) Qualitative patient interviews; 3) Consensus meeting.

There is likely some ability to reduce the budget in the involvement of the Research assistant/coordinator. Currently, the description describes a 1.0 FTE over 3 years. With the Graduate student leading Aim 1, and post-doctoral fellow leading the patient arm of the study, it is likely that the 1.0 FTE for the research staff can be substantially reduced to at least 0.5, if not even less. A 0.5 FTE would halve the $215,000 for this budget item.

Similarly, the post-doctoral fellow and graduate student amounts could be reduced from a full salary amount to a partial amount of support. Neither Aims 1 or 2 likely require a 1.0 FTE trainee to complete this, though the timeline for completion appears appropriate. Suggest reducing both of these budget requests to 0.5, reducing this budget item from $270,000 to $135,000.

Globally, the costs to complete Aim 3 are high also. Is it absolutely necessary to pay such a high rate of reimbursement and travel costs for all members of the panel?

**Review Type / Type d'évaluation:**	Reviewer 3 / Évaluateur 3
**Name of Applicant / Nom du chercheur:**	Sandal, Shaifali
**Application No. / Numéro de demande:**	526060
**Agency / Agence:**	CIHR/IRSC
**Competition / Concours:**	Project Grant/Subvention Projet
**Committee / Comité:**	Clinical Investigation - C: Digestive, Endocrine and Excretory Systems/Investigation clinique - C : Systèmes digestif, endocrinien et excrétoire
**Title / Titre:**	Disaster risk reduction and management in kidney care to foster health system resilience

Please indicate your appraisal of the integration of sex as a biological variable as a strength, weakness, or not applicable to the proposal./Prière de sélectionner une option pour donner votre évaluation de l'intégration du sexe comme variable biologique en tant que point fort ou point faible de la proposition, ou en tant qu'élément non applicable à la proposition.

- (●) **Strength/Point fort**
- ( ) **Weakness/Point faible**
- ( ) **Not applicable/Non applicable**

Please indicate your appraisal of the integration of gender as a socio-cultural determinant of health as a strength, weakness, or not applicable to the proposal./Prière de sélectionner une option pour donner votre évaluation de l'intégration du genre comme déterminant socioculturel de la santé en tant que point fort ou point faible de la proposition, ou en tant qu'élément non applicable à la proposition.

- (●) **Strength/Point fort**
- ( ) **Weakness/Point faible**
- ( ) **Not applicable/Non applicable**

**Review Type / Type d'évaluation:**	Reviewer 3 / Évaluateur 3
**Name of Applicant / Nom du chercheur:**	Sandal, Shaifali
**Application No. / Numéro de demande:**	526060
**Agency / Agence:**	CIHR/IRSC
**Competition / Concours:**	Project Grant/Subvention Projet
**Committee / Comité:**	Clinical Investigation - C: Digestive, Endocrine and Excretory Systems/Investigation clinique - C : Systèmes digestif, endocrinien et excrétoire
**Title / Titre:**	Disaster risk reduction and management in kidney care to foster health system resilience

Sex and/or Gender Considerations/Notions de sexe et/ou de genre:

As per the sex and/or gender section of the application, considerations are focused on aims 2 and 3. The investigators aim for a sex- and gender-balanced cohort for the consensus conference (aim 3). Analyses for aim 2 (qualitative work) will account for gender, but not sex, as the investigators believe gender to be more important in influencing participant experiences. They will set a minimum quota of 30% of participants being women, but will aim for gender-balanced participation, and will attempt to recruit gender-diverse individuals.

No specific concerns about this approach.

Review Type/Type d'évaluation: SO Notes /Notes de l'agent scientifique
Name of Applicant/Nom du chercheur: Sandal, Shaifali
Application No./Numéro de demande: 526060
Agency/Agence: CIHR/IRSC
Competition/Concours: 2024-09-11 Project Grant/Subvention Projet
Committee/Comité: Clinical Investigation - C: Digestive, Endocrine and Excretory Systems/Investigation clinique - C : Systèmes digestif, endocrinien et excrétoire
Title/Titre: Disaster risk reduction and management in kidney care to foster health system resilience

---

Assessment/Évaluation:

Strength

- Mid career NPI, junior FRQS application; transplant nephrologist, emerging scientist in climate and emergencies. Very well funded. Recent success.

- National network, international connections

- Knowledge users (nursing, administrators, patients) as stakeholder with LOS

- Identified need, original submission, well written proposal

- Super important topic

- Intention given to language and ethnicity representation

Weakness

- Improved environmental survey of existing disaster preparedness with explicit reporting of current players and shortcomings should be in background rather than Aim1?

- Feasibility: Greater detail of variables to be collected in Aim 1. Unsure sites will provide data as required.

- methods of aim2 (provide justification for population & speciality representation thresholds, geographic distribution)

- Applicability: Would disaster mitigation be better applied at the local level than national? Given provincial healthcare systems and local variability in disaster risk?

- Suitability of external validation from Australian healthcare system was questioned given goal is Canadian representation

- Large gap between Aims 1&2 and Aim #3. Greater detail of methods, objectives, deliverables needed for Aim #3. That said, we feel #3 is a laudable goal.

- Suggest including cyberattacks

- No specific inclusion of formal indigenous partner.

**Review Type/Type d'évaluation:**	SO Notes /Notes de l'agent scientifique
**Name of Applicant/Nom du chercheur:**	Sandal, Shaifali
**Application No./Numéro de demande:**	526060
**Agency/Agence:**	CIHR/IRSC
**Competition/Concours:**	2024-09-11 Project Grant/Subvention Projet
**Committee/Comité:**	Clinical Investigation - C: Digestive, Endocrine and Excretory Systems/Investigation clinique - C : Systèmes digestif, endocrinien et excrétoire
**Title/Titre:**	Disaster risk reduction and management in kidney care to foster health system resilience

Assessment/Évaluation:

SGBM

- Participant gender balance targets in Aim #2 & 3

Budget

- Overlap in roles of grad students and research assistants.
- Cut in research coordinator to 0.5 FTE reduced by $107,500 to $107,500
- Overlap in grad student and post-doc cost. Remove one of the roles. Reduce budget by $60,000.

Note: The final rating of the application, provided in the Notice of Recommendation (NOR) and Notice of decision (NOD), is the averaged rating of the peer review committee members following the discussion of the application during the committee meeting, and therefore may differ from the ratings provided by the assigned reviewers in their respective reviews.

Remarque : La cote définitive de la demande, qui apparaît dans l'avis de recommandation et l'avis de décision, représente la moyenne des cotes accordées par les membres du comité d'évaluation par les pairs après avoir débattu de la demande à la réunion du comité. Elle peut donc différer de celle donnée par les évaluateurs dans leur évaluation respective.
